# Development of the humeral head offset index for control of humeral torsion

**DOI:** 10.1186/s12891-024-07812-4

**Published:** 2024-09-09

**Authors:** Sam Razaeian, Julius Husarek, Sebastian Wangler, Cornelia L. A. Dewald, Ibrahim Al-Mousllie, Dafang Zhang

**Affiliations:** 1 Inselspital Bern, Department of Orthopaedic Surgery and Traumatology, Bern University Hospital, Freiburgstrasse 4, Bern, 3010 Switzerland; 2 Faculty of Medicine, Medical University of Sofia, Boulevard “Akademik Ivan Evstratiev Geshov” 15, Sofia, 1431 Bulgaria; 3 Institute for Diagnostic and Interventional Radiology, Hannover Medical School, Carl-Neuberg-Str. 1, 30625 Hannover, Germany; 4 Hannover Medical School, Carl-Neuberg-Str. 1, 30625 Hannover, Germany; 5 Department of Orthopaedic Surgery, Brigham and Women’s Hospital, 75 Francis St, Boston, MA 02115 USA; 6 Department of Trauma Surgery, Hannover Medical School, Carl-Neuberg-Str. 1, 30625 Hannover, Germany

**Keywords:** Torsional control, Retroversion, Torsion, Humeral shaft fracture; Proximal humerus fracture, Humeral head offset index

## Abstract

**Background:**

Control of humeral torsion can present a challenge, especially intraoperatively during closed reduction and fixation of humeral shaft fractures or 2-part surgical neck fractures of the proximal humerus. The objective of this study is to develop and validate an indirect method for the assessment of humeral torsion using an index that is linearly correlated with rotational arm position and can be derived from only a single plain radiographic image of the proximal humerus.

**Methods:**

The Humeral Head Offset Index (HHOI) is calculated as the ratio of the medial and lateral offset of the humeral head measured from the outer cortices of the shaft on a plain radiographic or fluoroscopic image. The relationship of HHOI with humeral torsion was first verified on a sawbone model with radiopaque characteristics under fluoroscopic control. Different degrees of retroversion were simulated through manual rotation of the humerus with a digital protractor in 5° increments until 40° internally rotated and then in 5° increments until 40° externally rotated from the neutral position. The same procedure was subsequently performed digitally on Digitally Reconstructed Radiographs (DRRs) from computed tomography (CT) dataset of the sawbone. Next, the HHOI index was applied to eight randomly selected patients with total humerus CT using the same method. Spearman’s rho was calculated for the bivariate analysis of correlation between the simulated degree of retroversion and the HHOI. Strength of correlation was classified according to Koo and Li. Interrater and intrarater reliability of three blinded observers with repetition of measurement after three months were analyzed by assessing the intraclass correlation coefficient (ICC).

**Results:**

Both in the sawbone model and in DRRs, we demonstrated a high to very high significant linear correlation between simulated retroversion and the HHOI. ICC values demonstrated excellent interrater reliability and excellent intrarater reliability for measurement of the HHOI.

**Conclusions:**

The HHOI is a new, simple, reliable index that has a linear relationship to the rotation of the humerus and can therefore allow an indirect control of humeral torsion in comparison to the contralateral side.

## Introduction

Control of humeral torsion can present a challenge, especially intraoperatively in the setting of a comminuted humeral shaft fracture (HSF) or a 2-part surgical neck fracture of the proximal humerus (PHF). Several anatomical, radiological, and sonographic measurement methods are known to determine humeral torsion [1, 2]. For intraoperative purposes, a previous study by Tan et al. has introduced a method using the position of the bicipital groove as a landmark under a fluoroscopic image intensifier for torsional control [3]. While this has been shown to be valid in human cadaveric models, in the setting of a surgically treated HSF or PHF, precise determination of the position of the bicipital groove might be difficult, for instance if there is overlay of radiopaque implants.

There is need for an easy, and reliable indirect method for the assessment of humeral torsion using plain radiograph of the proximal humerus, feasible even in the setting of a surgically treated HSF or PHF. The aim of this study is to develop the Humeral Head Offset Index (HHOI) in a radiopaque sawbones model and validate its correlation with humeral torsion in human patients with total humeral computed tomography. Our method might be applied intraoperatively during the surgical treatment of HSF or PHF using a single fluoroscopic image of the affected shoulder and a comparison fluoroscopic image of the contralateral unaffected shoulder to gauge torsional control.

## Materials and methods

This study was carried out in accordance with the ethical standards of the 1964 Declaration of Helsinki as updated in 2004. An approval of this study was given through the local ethical committee by waiver. In addition, an approved institutional data access agreement was obtained. Only patients who gave written informed consent for data usage for research purposes were retrospectively screened and included.

The motivation for this investigation is based on the observations of the first author (S.R.) that there may be an association between the mediolateral humeral head offset and rotational arm position. It has been observed by the first author that with increased internal rotation of the glenohumeral joint, the medial radiographic offset of the humeral head decreases while the lateral radiographic offset increases, while the reverse may be true for external rotation. The extent to which these assumptions are valid and reliable are examined in this study.

### Preliminary testing on sawbone

The association between mediolateral humeral head offset and humeral torsion was verified by the first author using a left humeral sawbone with radiopaque characteristics (Pacific Research Laboratories, Inc.Vashon Island, WA, United States). A conventional digital protractor (Junerain mini digital protractor, Shenzhen Si Hai Xin Zhou Technology Co Ltd., Shenzhen, China) was zeroed on the flat, horizontally positioned surface of the detector of an image converter (C-arm) (Ziehm Solo, Ziehm Imaging GmbH, Nürnberg, Germany). The protractor was fixed with its flat bottom anterior to the distal humerus at the level of an imaginary anterior trochlea tangent as described by Hernigou [4]. Afterwards the proximal humerus was rotated manually in 5° increments until 40° internally rotated and then in 5° increments until 40° externally rotated from the neutral position on the surface of the detector. An image was taken after each 5° increment of rotation.

**Fig. 1:**
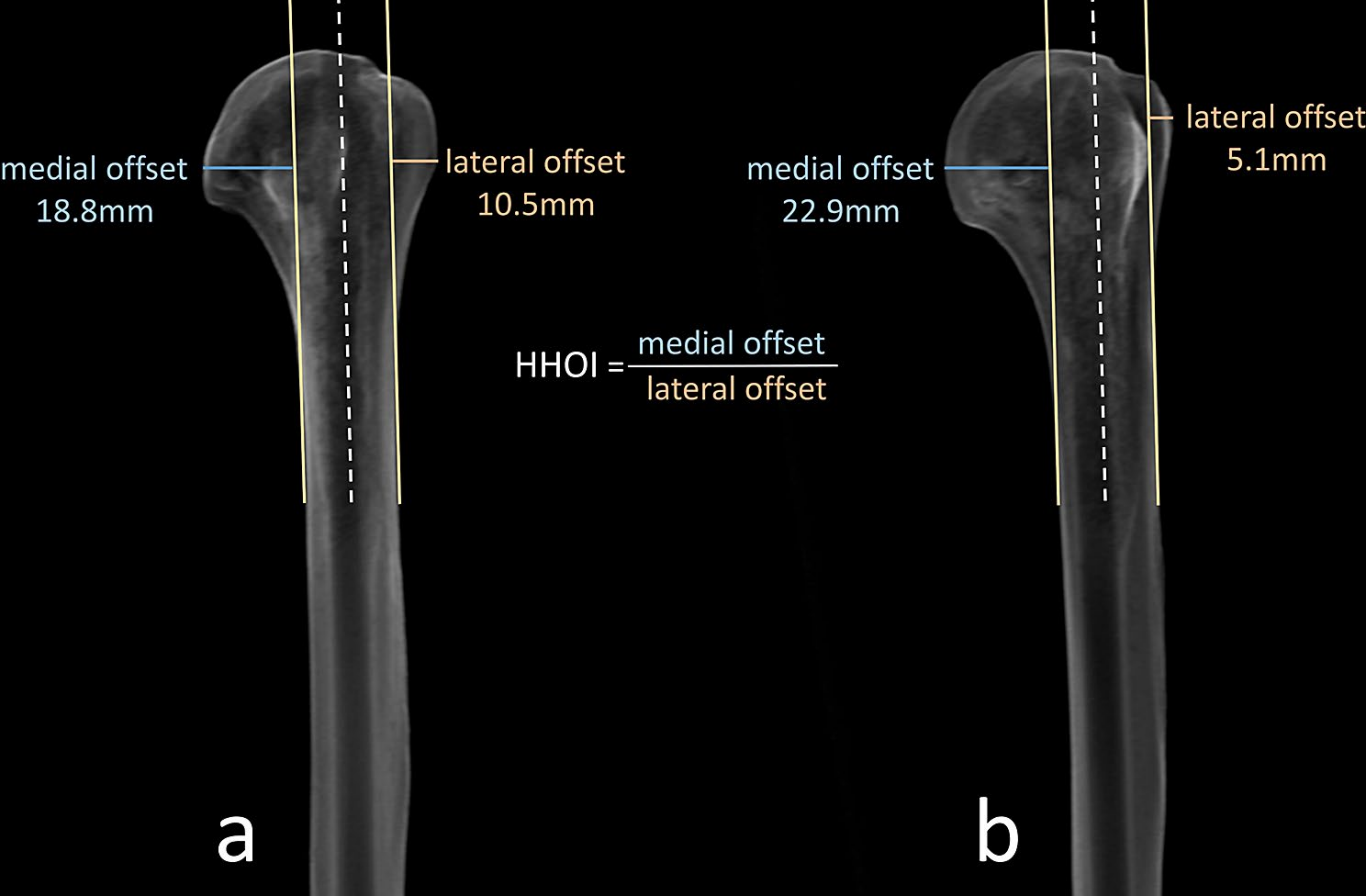
Simulated radiographic image of the sawbone in Average intensity (AvIP) mode of Visage 7.1 in (**a**) neutral position and (**b**) 40° of external rotation showing an appearance similar to that of a true anteroposterior radiograph of the shoulder. A tangent (yellow line) is drawn craniocaudally the proximal third of the humerus in the deltoid tuberosity region along the outer cortices parallel to the imaginary intramedullary axis (white line) and the medial offset, MO, (blue line) and lateral offset, LO, (red line) are measured

The Humeral Head Offset Index (HHOI) was calculated on these unreferenced images with picture archiving and communication software (PACS) Visage 7.1 (Visage Imaging Inc., San Diego, CA, United States). To calculate the HHOI, a craniocaudal line is drawn along the outer medial cortex of the proximal humerus (yellow line) parallel to the imaginary intramedullary axis (white line) (Fig. 1). The maximum perpendicular distance from the margin of the medial humeral head to this medial cortical line is defined as the medial offset (MO) (blue line). Next, a craniocaudal line is drawn along the outer lateral cortex of the proximal humerus (yellow line) parallel to the imaginary intramedullary axis (white line). The maximal perpendicular distance from the lateral margin of the humeral head to this lateral cortical line is defined as the lateral offset (LO) (red line). MO and LO can be measured in either millimeters or pixels. HHOI is defined as the quotient of these values (Fig. 1):

$$\:HHOI=\:\frac{MO}{LO}$$

Following calculation of HHOI from fluoroscopy, computed tomography (CT) of the same sawbone was obtained to verify the method. The true retroversion was calculated as described by Hernigou [4]. The previously selected anterior trochlear tangent for the manual fluoroscopic examination showed a deviation of only 1.4° of internal rotation in the CT examination compared to the transcondylar tangent, which is considered negligible by the authors for further calculations and comparisons. Then, Digitally Reconstructed Radiographs (DRRs) were made from the same CT dataset. For this purpose, in the multiplanar reconstruction mode (MPR) of Visage 7.1, the compositing mode for slices was changed to average intensity (AvIP) mode, and the thickness of slices was increased to 20 millimeters in order to simulate planar x-ray images (Fig. 1). Image axes were placed in the frontal and sagittal planes in the center of the medullary canal. In the axial plane, the crosshair and thus center of rotation was centered on the intersection of the head-neck axis and the transcondylar axis of previous retroversion measurement. At the beginning of the measurement, the humerus was rotated so that the frontal plane was parallel to the transepicondylar axis. In this way, a DRR was generated in AvIP mode that should correspond to that of an x-ray of the shoulder in the neutral rotation position, when the elbow joint is imagined to be 90° flexed and the x-ray beam is incident on the proximal humerus perpendicular to the transepicondylar axis, and parallel to the forearm axis (Fig. 1). From this starting position, the humerus was first rotated a total of 40° internally and then 40° externally in 5° increments. After every 5°, the HHOI was measured.

### Application in CT datasets of patients

To assess feasibility of this technique in patients, a retrospective search of CT records from total humeri was performed via the institutional Clinical Research Data Warehouse. All inpatients from January 1, 2015 to January 30, 2023 with available data use consent at a Level I trauma center were screened. CT imaging of the humerus, CT angiography imaging, and torsion differential CT imaging of the upper extremity in PACS were included. As any fracture of the proximal humerus or humeral shaft was an exclusion criterion, the following coded diagnosis according to the International Classification of Diseases – 10th Revision were used in order to filter these patients out: S42.20, S42.21, S42.22, S42.23, S42.24, S42.29, S42.3, S42.40, S42.41, S42.42, S42.43, S42.44, S42.45, and S42.49. Included patient records were anonymized by PACS accession number. One hundred twenty-eight CT datasets were provided, of which 37 had to be excluded after review. Five were excluded due to inserted endoprosthesis, 29 due to fractures, and 3 due to advanced glenohumeral arthrosis. Eight cases were randomly selected in order to apply the HHOI index. Since the humerus was first rotated a total of 40° internally and then 40° externally. Since the humerus was rotated a total of 80 degrees in 5° increments, 17 data points including the neutral position were obtained per each patient.

### Reliability analysis

A randomized sample of 30 blinded snapshots out of the CT dataset study pool was used to assess interrater reliability by one specialist of orthopedic trauma surgery (S.R.), one senior resident of radiology (C.D.), and one medical student (J.H.) with the PACS Visage 7.1 (Visage Imaging Inc., San Diego, CA, United States). The measurement was repeated after three months to assess intrarater reliability.

### Statistical analyses

For bivariate analysis of correlation between simulated degree of retroversion and medial offset, lateral offset, the mediolateral offset difference, and the HHOI, the correlation coefficient was calculated. Spearman’s rho (ρ) was used for nonparametric data. Correlation strength was classified as follows: very high: > 0.90; high: 0.70–0.89; moderate: 0.50–0.69; fair: 0.30–0.49; low: 0.10–0.29; or very low: 0.10.

Interrater reliability and intrarater reliability were analyzed by assessing the intraclass correlation coefficient (ICC). Strength of correlation was classified according to Koo and Li [5]. Values below 0.5 indicated poor reliability, between 0.5 and 0.75 moderate reliability, between 0.75 and 0.9 good reliability, and above 0.9 excellent reliability. A 95% confidence interval (CI) was set. P-values < 0.05 and < 0.01 were considered statistically significant and highly significant, respectively.

For the analyses, SPSS 26 (IBM, Armonk, New York) and Microsoft Excel 2016 (Microsoft Corporation, Redmond, Washington) were used.

## Results

### Preliminary testing on sawbone

**Fig. 2:**
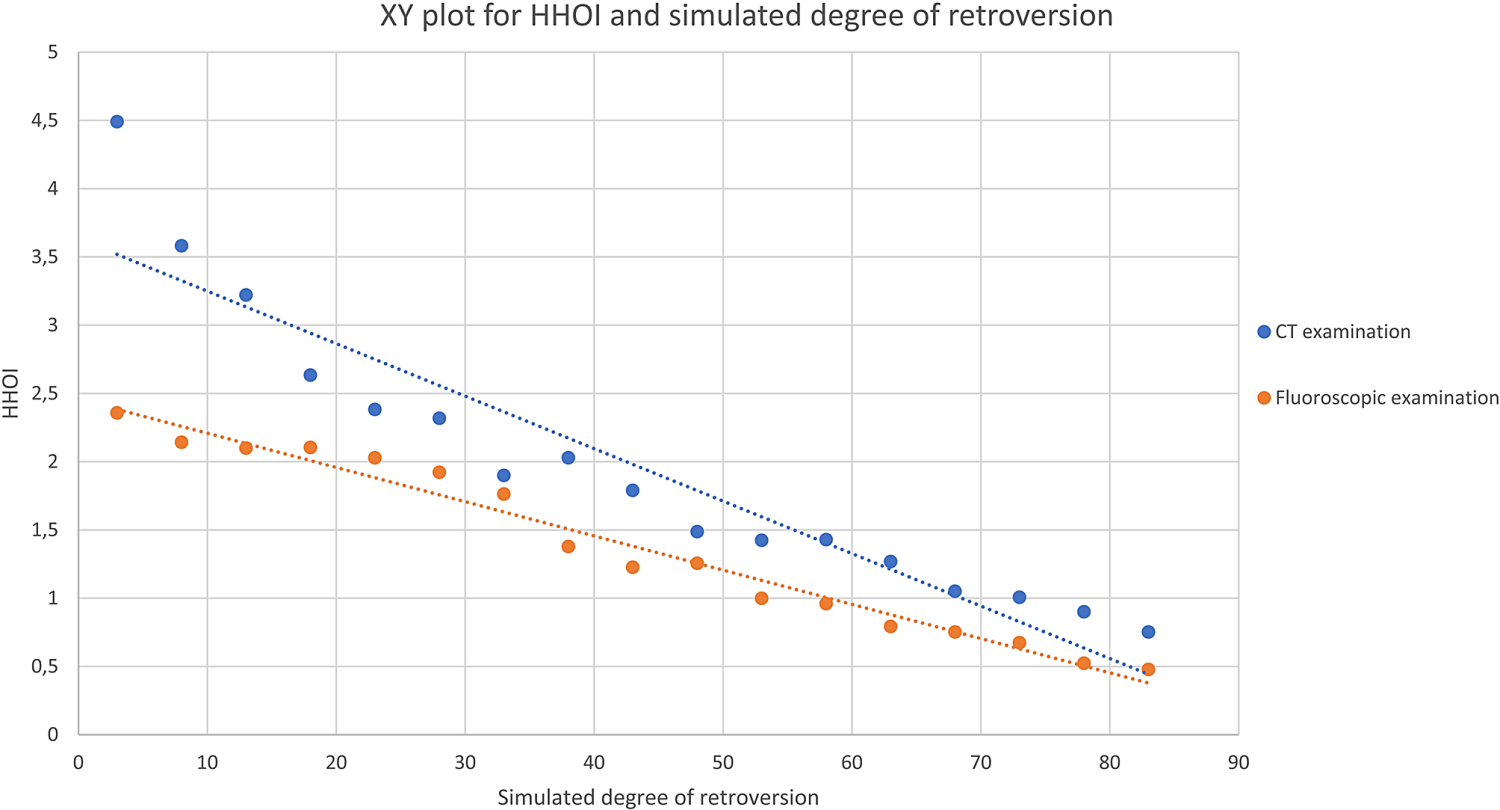
XY plot for HHOI and simulated degree of retroversion of sawbone model

**Fig. 3:**
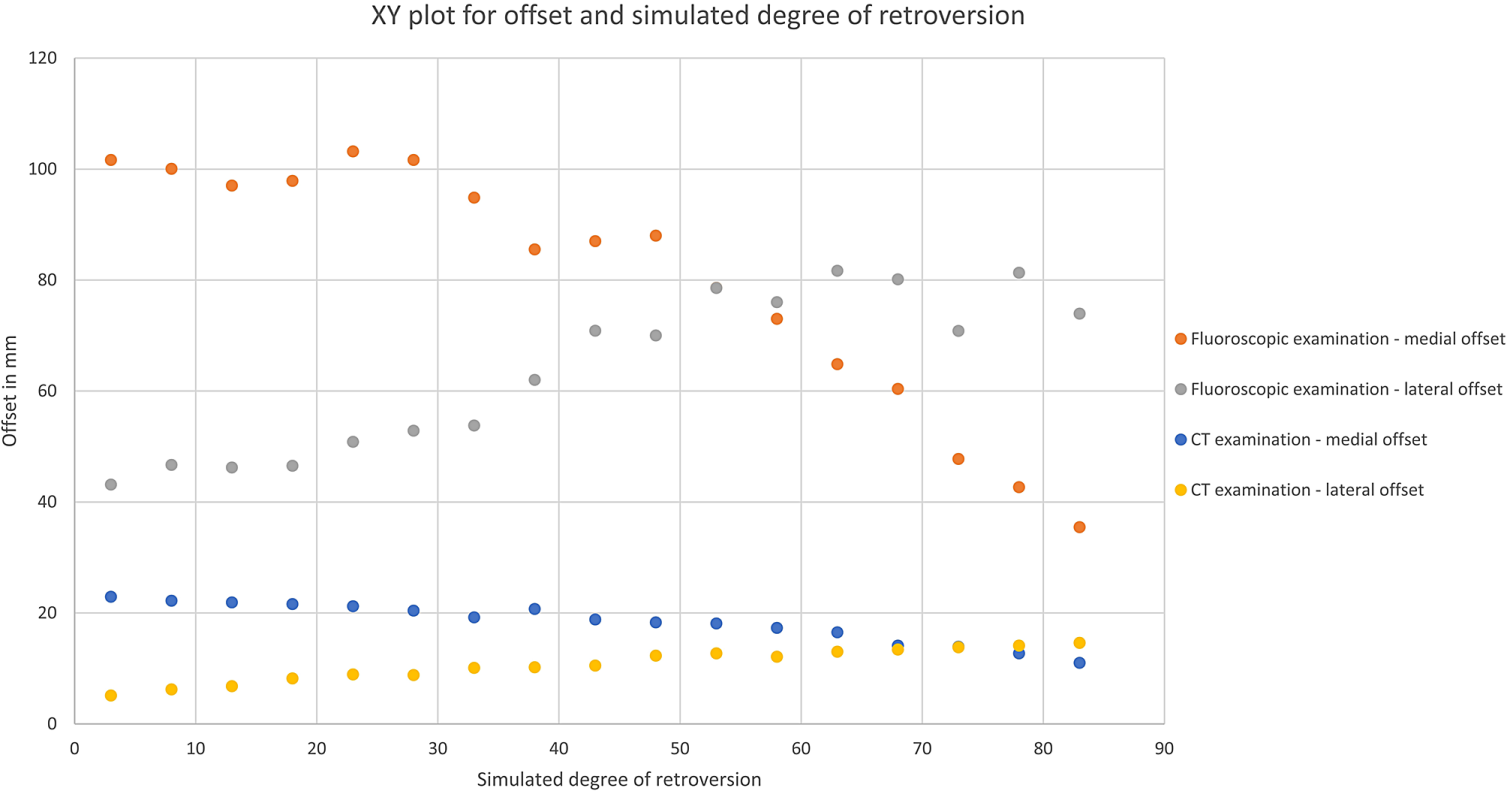
XY plot for mediolateral offset and simulated degree of retroversion of sawbone model

There was a very high significant negative correlation between simulated retroversion and the HHOI (Table 1; Fig. 2). As hypothesized, with increasing simulated retroversion through increased internal rotation, the medial offset decreased, while the lateral offset increased (Figs. 1 and 3). This relationship appeared to be linear, and this applied to both the manual fluoroscopic and digital CT examinations (Table 1; Figs. 2 and 3).

**Table 1:** Correlation coefficients (Spearman`s rho) for the relationship between simulated retroversion and measured parameters

	Examination mode	Medial offset in mm (95% CI)	Lateral offset in mm (95% CI)	Difference^1^ in mm (95% CI)	HHOI (95% CI)
**Simulated retroversion**	Fluoroscopic - sawbone	− 0.929** (-1 to − 0.73)	0.892** (0.61 to 0.99)	− 0.985** (-1 to − 0.91)	− 0.995** (-1 to − 0.96)
	CT - sawbone	− 0.993** (-1 to − 0.93)	0.99** (0.93 to 1)	− 0.998** (-1 to − 0.96)	− 0.995** (-1 to − 0.96)
	CT - patients	− 0.628** (-0.73 to − 0.51)	0.544** (0.41 to 0.66)	− 0.704** (-0.78 to − 0.61)	− 0.726** (-0.84 to − 0.64)

^1^ [Medial offset] – [lateral offset]. CI = confidence interval. ** p-value < 0.01

### Application in CT datasets of patients

In all eight patients, there was a significant negative correlation between simulated retroversion and the HHOI. The correlation was very high in six patients, and high in two patients (Table 2).

**Table 2:** Correlation coefficients (Spearman`s rho) for the relationship between simulated retroversion and measured parameters in each patient

Patient Number	Degree of retroversion^1^	Medial offset in mm (95% CI)	Lateral offset in mm (95% CI)	Difference^2^ in mm (95% CI)	HHOI (95% CI)
1	37	− 0.994** (-1 to − 0.95)	0.950** (0.77 to 1)	− 0.997** (-1 to − 0.96)	− 0.993** (-1 to − 0.93)
2	27	− 0.874** (-0.97 to − 0.61)	0.630** (-0.04 to 1)	− 0.785** (-1 to − 0.3)	− 0.978** (-1 to − 0.86)
3	42	− 0.800** (-0.98 to − 0.39)	0.729** (0.2 to 0.96)	− 0.815** (-1 to − 0.38)	− 0.784** (-0.98 to − 0.29)
4	40	− 0.980** (-1 to − 0.89)	0.956** (0.81 to 1)	− 0.99** (-1 to − 0.93)	− 0.99** (-1 to − 0.93)
5	14	− 0.856** (-1 to − 0.46)	0.929** (0.75 to 0.99)	− 0.915** (-0.99 to − 0.62)	− 0.926** (-1 to − 0.67)
6	19	− 0.549* (-0.94 to 0.02)	0.853** (0.59 to 0.95)	− 0.696** (-1 to − 0.23)	− 0.843** (-0.99 to − 0.53)
7	40	− 0.953** (-1 to − 0.77)	− 0.16 (-0.86 to 0.52)	− 0.982** (-1 to − 0.9)	− 0.971** (-1 to − 0.85)
8	33	− 0.882** (-0.98 to − 0.58)	0.04 (-0.52 to 0.59)	− 0.985** (-1 to − 0.89)	− 0.975** (-1 to − 0.86)

^1^ CT-based measurement according to Hernigou. ^2^ [Medial offset] – [lateral offset]. CI = confidence interval. * p-value = 0.031 ** p-value < 0.01

**Fig. 4:**
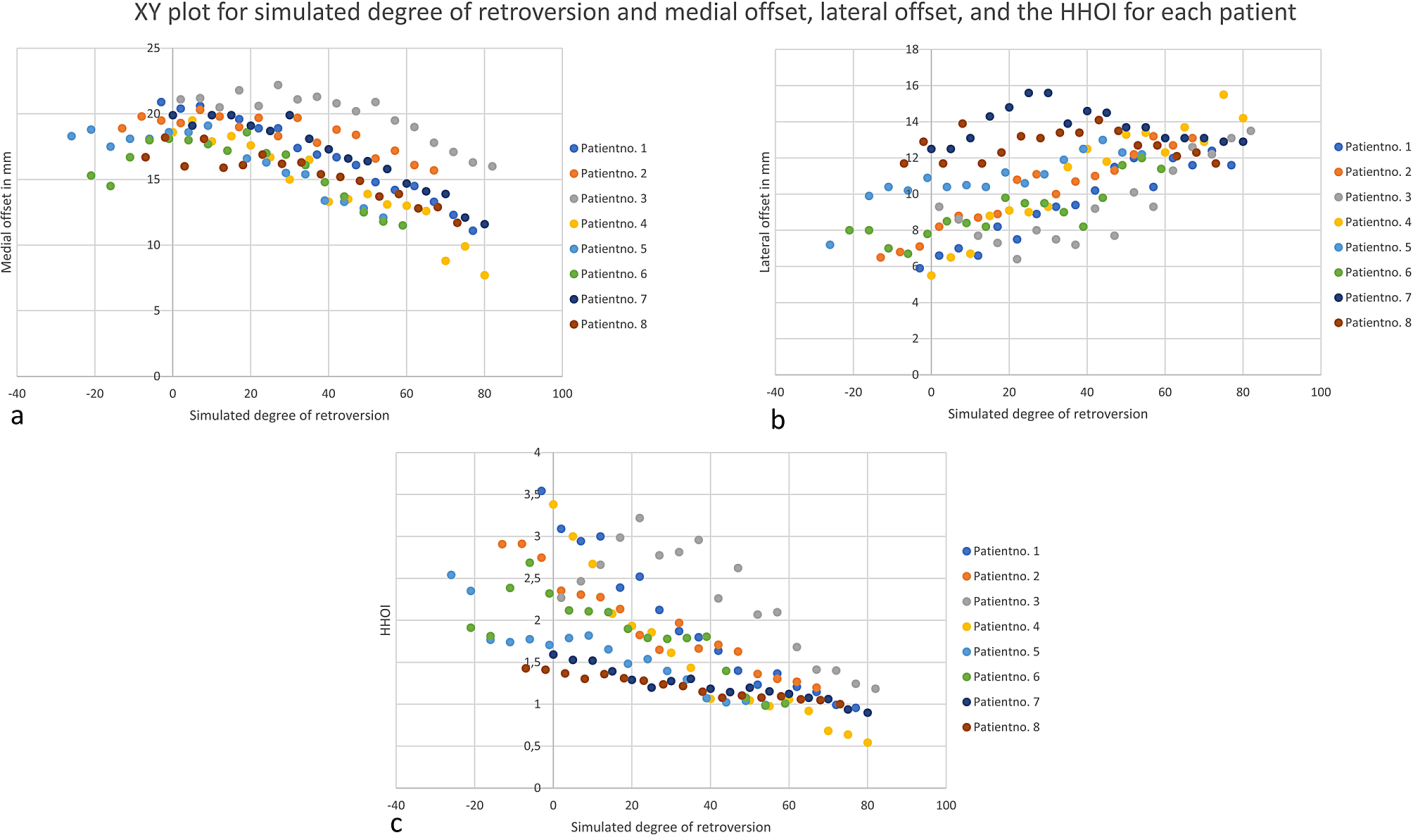
XY plot for simulated degree of retroversion and medial offset (**a**), lateral offset (**b**), and the HHOI (**c**) for each patient

Figure 4 shows the relationship between simulated retroversion and the HHOI, medial offset, and lateral offset for each patient. Pooled data of all eight patients also revealed a high negative correlation between simulated retroversion and the HHOI (Table 1).

ICC values demonstrated excellent interrater reliability (0.97, 95% CI, 0.94–0.98) and excellent intrarater reliability (0.98, 95% CI, 0.95–0.99; 0.92; 95% CI, 0.83–0.96; 0.98; 95% CI, 0.99–0.99) for measurement of the HHOI.

## Discussion

### Principal findings

Measurement of humeral torsion remains a clinical challenge, and there is not an agreed upon intraoperative method of humeral torsional control when surgically treating a HSF or PHF [3]. Humeral torsion can be measured by conventional radiography, computed tomography, magnetic resonance imaging, or ultrasonography. Computed tomography has the disadvantage of additional radiation exposure, magnetic resonance imaging the disadvantage of increased cost and metal-induced artifacts, and ultrasonography can be practically difficult to apply intraoperatively [1]. The motivation of this study was to develop and validate an indirect measurement of humeral torsion that relies on only a single radiographic image, that can be readily applied intraoperatively, and that has reliability within and between observers. The HHOI relies on the predictable decrease in radiographic medial offset of the humeral head and increase in radiographic lateral offset of the humeral head with glenohumeral joint internal rotation, and vice versa with external rotation. In this study, we have proved the concept of the HHOI preliminarily on a sawbone model prior to validation in eight patients using CT humeri as the reference standard for humeral torsion. We have demonstrated high to very high correlation between HHOI and simulated retroversion based on CT in all tested patients. Finally, we have demonstrated excellent interrater and intrarater reliability for measurement of the HHOI.

Our findings have practical applications, for instance, for control of humeral torsion intraoperatively in the surgically treatment of HSF, particularly in the setting of comminuted HSF where the surgical goals are restoration of length, alignment, and rotation. In these cases, judgment of humeral torsion is generally indirect and yet may be important for patients’ ultimate outcomes. We believe that the HHOI can be a useful intraoperative tool for the judgment of humeral torsion, particularly in cases of proximal humeral fractures with metadiaphyseal comminution, where confirmation of rotational alignment through a direct reduction may be unavailable. In a recent study of humeral torsional side differences among patients with nonoperatively treated proximal humerus fracture and HSF, we showed that most humeral torsional side differences were less than 15° and subjectively imperceptible. However, torsional side differences greater than 30° were more likely to be perceived by patients and showed statistically significant correlation with poorer passive shoulder range of motion and worse subjective shoulder value (SSV) [6]. In a retrospective clinical study of 15 patients treated with retrograde nailing and 15 patients treated with antegrade nailing for closed humerus fractures, Lin and Hou showed that rotational malalignment of the arm may be affected by intraoperative positioning, but the authors commented on the lack of a reliable intraoperative method of measuring humeral rotation [7]. Our results show promise that intraoperative measurement of the HHOI can be valuable in preventing humeral malrotation in this common clinical scenario. Our method should be contrasted with other existing methods for the intraoperative judgment of humeral torsion. Most existing methods rely on the use of the bicipital groove, but radiographic visualization of the bicipital groove may be obscured by an intramedullary implant, such as a humeral nail. Meriç et al. published a cadaveric study of 30 upper extremities, describing utilization of the bicipital groove axis by clinical visualization to judge humeral rotation [8]. Disadvantages of this technique include the lack of radiographic correlation, the potential variability of the relationship of the bicipital groove axis and the transepicondylar axis among different patients, and the lack of a contralateral comparison. Furthermore, in proximal humeral fractures with comminution of the lesser and/or greater tuberosities, the anatomic borders of the bicipital groove may be distorted or unavailable for reference. The HHOI is an easy and reliable method of judging humeral torsion, using a single fluoroscopic image of the affected shoulder, which can be compared against a single fluoroscopic image of the contralateral unaffected shoulder.

As we have demonstrated a strong linear correlation between the HHOI and rotational arm position, intraoperative measurement, and comparison against the contralateral unaffected side serving as a reference may be useful to gauge, and adjust humeral torsion in a similar manner that is established for torsional control during closed treatment of femoral shaft fractures by using the profile of the contralateral lesser trochanter [9–12]. 

We believe that a radiolucent custom-made ruler could assist to directly read and compare already assigned indices.

### Limitations

This study has several limitations that need to be considered. Firstly, we have introduced only an indirect method of measuring humeral torsion, similar to the well-known lesser trochanter method for torsional control of the femur, based on the assumption of anatomical equality of the bony anatomy of bilateral sides. Although we have observed a promising coherence among different individuals, our study lacks an analysis of the index applied on both sides of the same individual. Since a strong to very strong significant linear correlation had already been demonstrated in the preliminary testing, we calculated that a randomly selected sample of only 8 patients was sufficient to confirm this correlation, which ultimately proved to be the case. Nevertheless, this small number of cases must be seen as a limitation, as the generalizability and statistical power are margined.

Furthermore, the ease and reliability of the practical implementation of the HHOI intraoperatively remains unclear.

## Conclusion

The HHOI is a new, simple, reliable index that has a linear relationship to the rotation of the humerus and can therefore allow an indirect control of humeral torsion in comparison to the contralateral side.

## Data Availability

Raw data will be made available on request.
